# Feasibility and short-term outcomes of transcatheter closure of moderate-to-large perimembranous ventricular septal defects in children using bioabsorbable occluders: a prospective case series

**DOI:** 10.3389/fcvm.2026.1930121

**Published:** 2026-08-24

**Authors:** Mai Chen, Liuliu Huang, Decai Zeng, Chunlan Jiang, Guoxing Ling, Ji Wu, Baoshi Zheng

**Affiliations:** 1 Department of Cardiac Surgery, The First Affiliated Hospital of Guangxi Medical University, Nanning, China; 2 Department of Echocardiography, The First Affiliated Hospital of Guangxi Medical University, Nanning, China

**Keywords:** bioabsorbable occluder, children, moderate-to-large ventricular septal defect, perimembranous ventricular septal defect, transesophageal echocardiography

## Abstract

**Objectives:**

Transcatheter closure has become an important treatment modality for perimembranous ventricular septal defects (pmVSDs). However, conventional metallic occluders carry the risk of long-term complications. Fully bioabsorbable occluders offer a potential solution, yet their application in children with moderate-to-large pmVSDs remains under-reported. This study evaluated the feasibility and 1-year outcomes of transthoracic, transesophageal echocardiography (TEE)-guided implantation of a bioabsorbable occluder via a small subxiphoid incision in children with moderate-to-large pmVSDs.

**Methods:**

Children aged ≥1 year with moderate-to-large pmVSDs (defined as left ventricular defect diameter exceeding one-third of the aortic root diameter) who underwent TEE-guided transthoracic device closure using a bioabsorbable occluder between July 2024 and January 2025 were prospectively enrolled. Baseline characteristics, procedural data, and follow-up outcomes at 1, 3, 6, and 12 months were collected. The primary endpoints were technical success, valvular function, conduction disturbance, and occluder degradation.

**Results:**

Five children (mean age 2.4 ± 1.7 years; mean weight 12.6 ± 4.6 kg; mean defect diameter 8.8 ± 2.6 mm) were included. All implants were successful (technical success 100%). One patient with a multi-fenestrated defect had a 2 mm residual shunt immediately post-procedure, which resolved by 6 months. At 1 year, no patient developed new-onset or worsening aortic regurgitation (AR) or tricuspid regurgitation (TR), and none had complete atrioventricular block (cAVB). Quantitative echocardiographic analysis showed that occluder disc area decreased substantially at 1 year, indicating progressive absorption.

**Conclusions:**

TEE-guided transthoracic closure of moderate-to-large pmVSDs using a bioabsorbable occluder appears feasible in selected children. One-year follow-up data suggest favorable preservation of valvular and conduction function, with progressive occluder degradation. This approach may offer a minimally invasive alternative to surgery for this challenging population, although larger studies are warranted.

## 1 Introduction

Perimembranous ventricular septal defects (pmVSDs) are the most common type of ventricular septal defect (VSD), accounting for 70%–80% of all VSDs (1). Although surgical repair has long been the gold standard, it carries inherent drawbacks including surgical trauma, cardiopulmonary bypass requirement, and visible scarring. Since the first transcatheter pmVSD closure in 2002, this minimally invasive approach has gained wide acceptance owing to its faster recovery and reduced procedural morbidity (2, 3). However, concerns regarding the long-term safety of nitinol occluders—particularly delayed atrioventricular block—have persisted, preventing regulatory approval for pmVSD closure in some countries (4–6).

Fully bioabsorbable occluders offer a promising alternative. Constructed from a polydioxanone (PDO) framework and poly-L-lactic acid (PLLA) flow-reducing membrane, these devices gradually hydrolyze to carbon dioxide and water after serving their sealing function, thereby eliminating the risks associated with permanent metallic implants (7, 8). Chen et al. (9) first reported human implantation of a fully bioabsorbable VSD occluder. Subsequently, Wang et al. (10), in a multicenter randomized controlled trial (RCT), demonstrated non-inferiority of bioabsorbable occluders compared with nitinol occluders, with a lower incidence of sustained conduction block. Song et al. (11) and Cong et al. (12) further confirmed favorable early-to-mid-term outcomes.

Notably, the defect size in these studies was predominantly small (median diameter 4.0–5.1 mm). For moderate-to-large pmVSDs—particularly in young children—interventional closure remains challenging. Such defects require larger occluders and are often associated with more complex anatomy, increasing the risk of compression on adjacent structures including the aortic valve, tricuspid valve, and conduction system. Pillai et al. (13) reported a technical success rate of 85.7% for metallic occluder closure of moderate-to-large pmVSDs in children ≤10 kg, with a non-negligible rate of valve regurgitation and conduction abnormalities. To date, no study has specifically evaluated bioabsorbable occluders in this high-risk subgroup.

Building on our center’s extensive experience in ultrasound-guided interventional procedures for congenital heart disease, we report here the feasibility and 1-year outcomes of TEE-guided transthoracic closure of moderate-to-large pmVSDs using a bioabsorbable occluder in children.

## 2 Materials and methods

### 2.1 Study design and patient population

This was a single-center, prospective case series. Consecutive children with moderate-to-large pmVSDs who underwent bioabsorbable occluder implantation at our center between July 2024 and January 2025 were enrolled.

#### 2.1.1 Inclusion criteria

(1) age ≥1 year; (2) isolated pmVSD confirmed by transthoracic echocardiography (TTE); (3) moderate-to-large defect, defined as defect diameter exceeding one-third of the aortic root diameter; (4) left-to-right shunt with indication for intervention; and (5) informed consent provided by legal guardians.

#### 2.1.2 Exclusion criteria

(1) concomitant cardiac anomalies requiring surgical correction; (2) moderate-to-severe aortic valve prolapse; (3) pre-existing moderate or greater aortic regurgitation (AR) or tricuspid regurgitation (TR); (4) malalignment pmVSD; (5) severe pulmonary hypertension with bidirectional or right-to-left shunt (Eisenmenger syndrome); (6) active infective endocarditis or recent severe infection; (7) contraindications to antiplatelet therapy; and (8) pre-existing atrioventricular conduction block.

### 2.2 Echocardiographic assessment and grading criteria

#### 2.2.1 Preoperative assessment

Comprehensive TTE (Philips iE33 or EPIQ 7C, Philips Medical Systems, Andover, MA, USA) was performed by an experienced echocardiographer. The following parameters were assessed: defect location, size, morphology, shunt direction, and spatial relationship to surrounding structures. Defect base diameter and distance from the upper defect margin to the aortic annulus were measured. AR, TR, and left ventricular (LV) dimensions and function were also evaluated.

#### 2.2.2 Grading of valve regurgitation and residual shunt

AR severity was assessed by the ratio of regurgitant jet width to LV outflow tract width (14). TR was graded according to established guidelines (15). Residual shunt was classified by color Doppler jet width as trivial (<1 mm), small (1–2 mm), moderate (2–4 mm), or large (>4 mm) (16).

### 2.3 Bioabsorbable occluder

The fully bioabsorbable VSD occluder (Shanghai Shape Memory Alloy Co., Ltd., China) is woven from PDO monofilament into a double-disc frame with an inner PLLA membrane. The PDO framework begins active degradation approximately 90 days post-implantation, while the PLLA membrane degrades more slowly to maintain structural integrity during the critical healing phase (9). Four waist-height configurations are available (types I–IV, with waist heights of 2.8, 5, 7, and 10 mm, respectively), each offering waist diameters of 4–16 mm. Disc diameter is 5 mm larger than waist diameter (2.5 mm on each side). Delivery sheath sizes range from 7 to 10 F. The PDO material has a lower elastic modulus than nitinol; therefore, a shaping line is required to assist in forming the double-disc configuration during deployment. The occluder remains retrievable and redeployable until the shaping line is fully tightened.

### 2.4 Surgical procedure

All procedures were performed in a hybrid operating room under general anesthesia with TEE guidance, with cardiopulmonary bypass on standby. TEE (Philips X7-2t or GE 6VT-D) was used to assess the defect in multiple planes (four-chamber, five-chamber, short-axis, and long-axis views). The maximum defect base diameter (typically measured in the short-axis view) guided occluder size selection. Based on prior experience, the occluder was sized 1–2 mm larger than the defect diameter. For defects close to the tricuspid septal leaflet, type I (short waist) was preferred to minimize interference with tricuspid apparatus. For defects adjacent to the aortic valve, type II (medium waist) was selected to increase the spatial distance between the occluder disc and the aortic valve. Types III (7 mm) and IV (10 mm) are available for adults or more complex anatomies.

A 2–3 cm subxiphoid incision was made, and the lower sternum was partially split (2–3 cm). The pericardium was opened to expose the right ventricular (RV) free wall. Under TEE guidance, a puncture site perpendicular to the interventricular septum was selected on the RV anterior wall, avoiding major coronary arteries. A 5-0 or 4-0 Prolene purse-string suture with a pledget was placed. After systemic heparinization (100 IU/kg), an 18-G cannula was introduced at the puncture site. A 0.035-inch guidewire was advanced through the cannula, across the pmVSD, and into the LV under TEE guidance. The delivery sheath was then advanced over the guidewire into the LV cavity (avoiding the mitral valve chordae), and the dilator and guidewire were removed.

**Figure 1:**
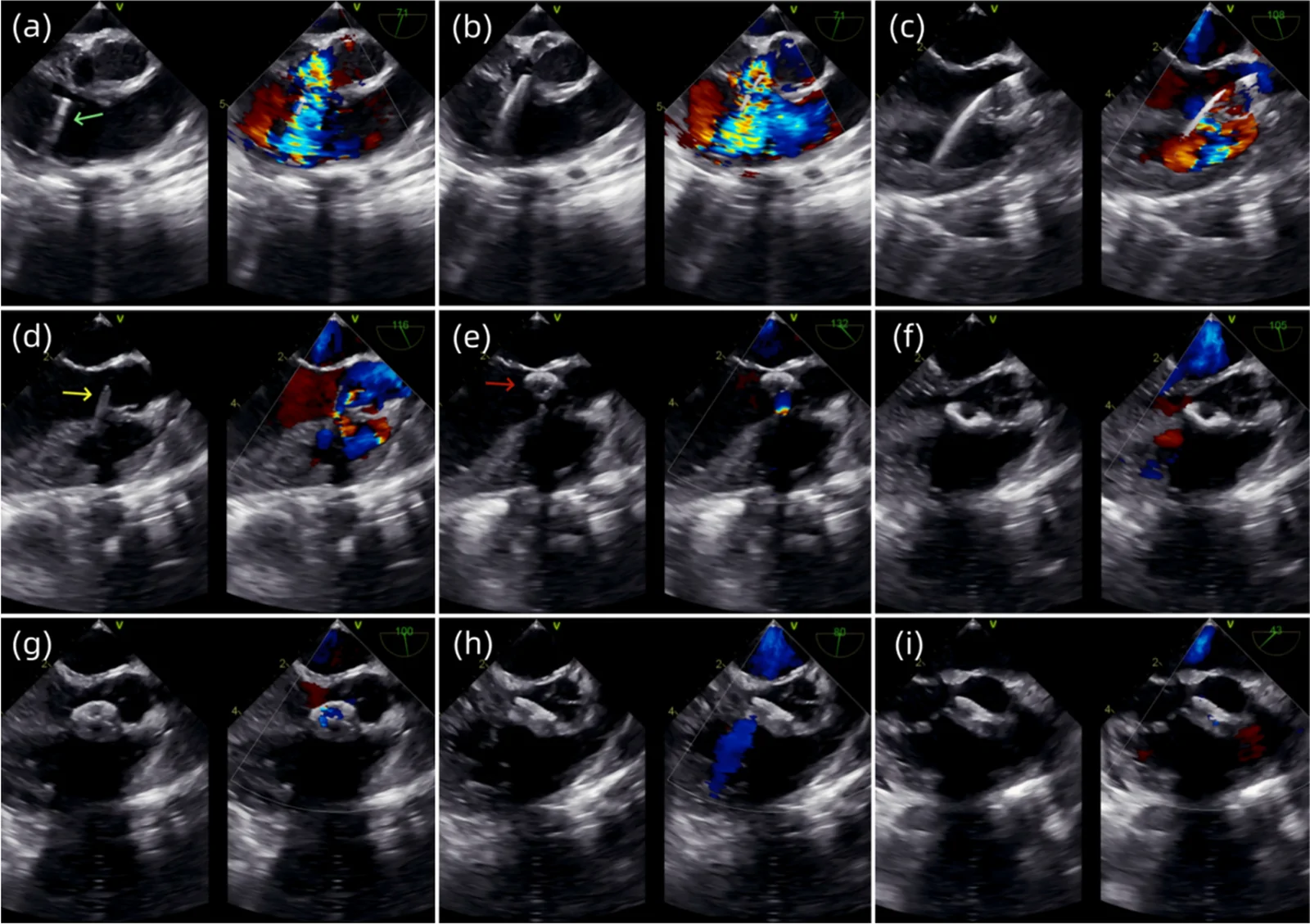
Transthoracic implantation of the bioabsorbable occluder under TEE guidance. **(a–i)**: **(a)** RV free-wall puncture and guidewire (green arrow) advancement. **(b)** Guidewire crossing the pmVSD from the RV side. **(c)** Guidewire tip positioned in the ascending aorta (parasternal long-axis view). **(d)** Delivery sheath (yellow arrow) advanced into the LV. **(e)** Left disc deployment (red arrow). **(f)** Left disc retracted against the septum. **(g)** Waist and right disc deployment. **(h)** Final locked occluder with no AR. **(i)** No TR on short-axis view. LV, left ventricle; RV, right ventricle; AR, aortic regurgitation; TR, tricuspid regurgitation; pmVSD, perimembranous ventricular septal defect.

The pre-loaded occluder was delivered through the sheath to the LV. Under real-time TEE guidance, the left disc was deployed (initially assuming a spherical shape), and the shaping line was pulled to transform it into a flat disc apposed to the LV septal surface. The delivery sheath and cable were withdrawn to bring the left disc against the septum, followed by deployment of the waist and right disc. The shaping line was then fully tightened to lock the occluder into the final double-disc configuration. Final assessment with TEE confirmed adequate position, morphology, residual shunt (≤2 mm was considered clinically acceptable), and normal aortic and tricuspid valve function. A push-pull test was performed to verify stability. The occluder was released by counter-clockwise rotation of the delivery cable. The delivery system was withdrawn, and the purse-string suture was tied. All patients were transferred to the intensive care unit for early extubation. Representative procedural steps are shown in Figure 1.

### 2.5 Postoperative management and follow-up

Aspirin (3–5 mg/kg/day) was administered for 6 months postoperatively. Follow-up visits were scheduled at 1, 3, 6, and 12 months, with clinical evaluation, 12-lead electrocardiography (ECG), and TTE performed at each visit.

### 2.6 Quantitative analysis of occluder degradation

To quantify *in vivo* degradation, the hyperechoic areas corresponding to the left and right discs were measured in the standard apical five-chamber, parasternal short-axis, and parasternal long-axis views, as previously described (10). The region of interest was delineated using QLAB (Philips Medical Systems) or EchoPAC (GE Healthcare) software. Area measurements were compared between immediate post-implant and follow-up time points (1, 3, 6, and 12 months).

### 2.7 Statistical analysis

Given the limited sample size, descriptive statistics were primarily used. Continuous variables are expressed as mean ± standard deviation or median (range). Categorical variables are presented as frequencies and percentages.

## 3 Results

### 3.1 Baseline characteristics

A total of five children (three females) were enrolled. Mean age was 2.4 ± 1.7 years (range: 1.0–5.0 years), and mean weight was 12.6 ± 4.6 kg (range: 8.0–19.0 kg). Mean LV defect diameter was 8.8 ± 2.6 mm (range: 5.0–12.0 mm). One patient had a multi-fenestrated defect (two RV exit openings, 6 mm and 2 mm), two had a subaortic rim <3 mm, three had defects in proximity to the tricuspid septal leaflet, and two had mild TR. All patients had sinus rhythm with no bundle-branch block preoperatively. Baseline characteristics are summarized in Table 1.

**Table 1:** Baseline characteristics and procedural data.

Case	Age, year	Weight, kg	VSD Size, mm	LVEDD, mm	AV prolapse	SAR < 3mm	Adjacent to TV	Occluder	Delivery sheath	Residual shunt, mm		New-onset AR		New-onset TR		New-onset conduction disturbance	
										Intraop	FU	Intraop	FU	Intraop	FU	Intraop	FU
1	3.0	15.8	10.0	40.0	None	N	Y	II-12→I-12	9F	0.0	0.0	None	None	None	None	None	None
2	2.0	10.0	9.0	35.0	None	N	Y	I-10	9F	0.0	0.0	None	None	None	None	None	None
3	5.0	19.0	12.0	42.0	None	N	Y	I-14	10F	0.0	0.0	None	None	None	None	IRBBB^a^	None
4	1.0	8.0	8.0	27.0	mild	Y	N	II-10→II-9	10F	2.0^b^	0.0	None	None	None	None	None	None
5	1.0	9.0	5.0	34.0	None	Y	N	II-6	8F	0.0	0.0	None	None	None	None	None	None

VSD, ventricular septal defect; LVEDD, left ventricular end-diastolic diameter; AV, aortic valve; SAR, subaortic rim; TV, tricuspid valve; F, French gauge; Intraop, intraoperative; FU, follow-up; AR, aortic regurgitation; TR, tricuspid regurgitation.

a IRBBB, incomplete right bundle branch block (transient, resolved at 3-month follow-up).

b A mild (2 mm) residual shunt detected immediately after implantation in Case 4 resolved completely by the 6-month follow-up.

### 3.2 Procedural outcomes

Device implantation was successful in all five patients (100% technical success rate). Mean procedural time was 98.0 ± 24.1 min, mean blood loss was 26.0 ± 5.5 mL, and mean occluder diameter was 10.2 ± 3.1 mm (range: 6.0–14.0 mm). In case 1, initial use of a type II-12 occluder resulted in increased TR; this resolved after replacement with a type I-12 occluder (shorter waist). In case 4 (subaortic rim <3 mm), trial of a type II-10 occluder provoked mild AR, which resolved after downsizing to a type II-9 occluder. This patient also had a multi-fenestrated defect, with a 2 mm residual shunt observed immediately after implantation. No deaths, occluder embolization, cardiac perforation, or pericardial tamponade occurred. Mean postoperative hospital stay was 3.4 ± 0.6 days (range: 3.0–4.0 days). Procedural data are detailed in Table 1.

### 3.3 Follow-up outcomes

All patients completed the 12-month follow-up (100% follow-up rate).

#### 3.3.1 Valvular function, conduction, and residual shunt

At 1 year, no patient developed new-onset or worsening AR or TR (Table 1). Two patients with pre-existing mild TR (cases 1 and 3) showed improvement. All patients remained in sinus rhythm, and no complete atrioventricular block (cAVB) occurred. One patient (case 3) had transient incomplete right bundle-branch block (IRBBB) that normalized by 3 months. No left bundle-branch block or other arrhythmias were observed. The 2 mm residual shunt in case 4 decreased to 1 mm at 1 month, became trivial (<1 mm) at 3 months, and resolved completely by 6 months. No residual shunt was detected in other patients.

#### 3.3.2 Occluder degradation

**Figure 2:**
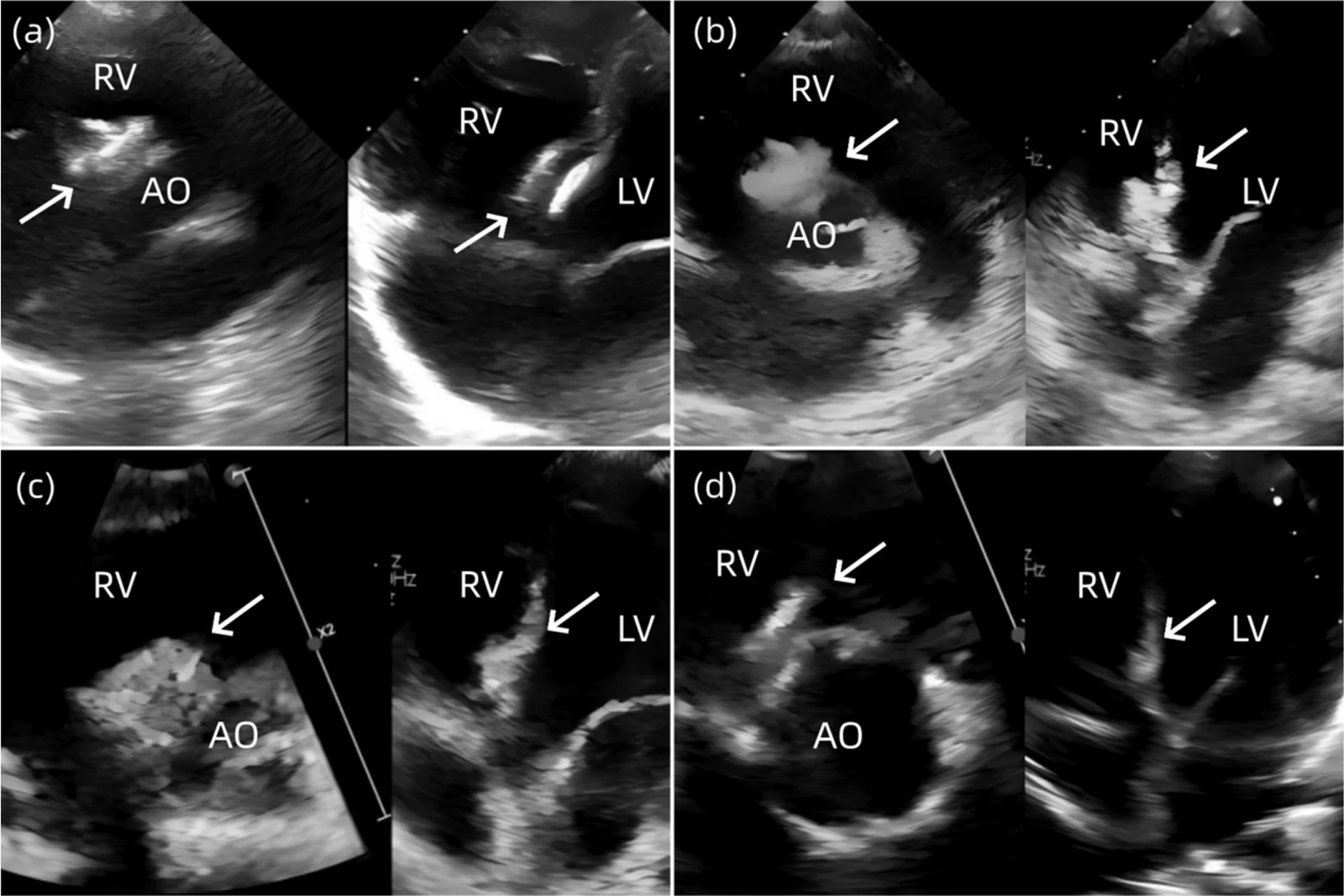
Serial echocardiographic changes of the bioabsorbable occluder at 1-year follow-up. **(a–d)**: TTE short-axis view and five-chamber view were examined at 1-, 3-, 6-, and 12-month follow-up, respectively. Arrows indicate the occluder. AO, ascending aorta; RV, right ventricle; LV, left ventricle.

At 1 year, the occluder appeared as a blurred hyperechoic area blending with the surrounding endocardium. Quantitative analysis showed that left disc area decreased from a mean of 1.68 ± 0.73 cm^2^ (immediate post-implant) to 0.25 ± 0.06 cm^2^ (1 year), representing a reduction of 82.6%. Right disc area decreased from 1.75 ± 0.79 cm^2^ to 0.39 ± 0.16 cm^2^, representing a reduction of 76.0%, suggesting a trend toward faster left-disc degradation. Serial echocardiographic changes are illustrated in Figure 2.

## 4 Discussion

This pilot study reports preliminary experience with bioabsorbable occluders for moderate-to-large pmVSDs in children. All five implants were successful, with no deaths, embolization, or tamponade, confirming feasibility even in anatomically complex cases. At 1-year follow-up, no patient developed VSD recurrence, new-onset or worsening aortic or tricuspid regurgitation, or cAVB. Serial echocardiography showed that the occluder disc area decreased progressively over time, indicating gradual device absorption.

The technical success rate (100%) compares favorably with previously reported series. A meta-analysis of perventricular pmVSD closure reported a pooled success rate of 95% (17). The mean defect diameter in our cohort (8.8 mm) was substantially larger than in earlier bioabsorbable occluder studies (median 4.0–5.1 mm) (9, 10), representing a more challenging population. Pillai et al. (13) reported a success rate of 85.7% for moderate-to-large pmVSDs using metal occluders, with failures attributed to device slippage, difficulty in track establishment, and intraoperative cAVB. The shorter, more direct delivery pathway in our transthoracic approach may have contributed to the higher success rate. The 2 mm residual shunt observed in one patient resolved by 6 months, consistent with reports that most residual shunts close spontaneously (17, 18).

All patients underwent a subxiphoid, right ventricular free-wall puncture approach. This route offers several advantages. Moderate-to-large pmVSDs require large occluders and correspondingly large delivery sheaths (up to 10F in this series). Bioabsorbable occluders require sheaths 1–2F larger than metal occluders of the same nominal size, due to the need to maintain structural integrity during passage (10). In young, low-weight children, transfemoral access carries risk of vessel-size mismatch and vascular injury (19); transthoracic RV puncture circumvents these limitations. The short, direct delivery path also facilitates precise axial adjustment, which is particularly beneficial in complex anatomies. Compared with the percutaneous route, the transthoracic approach is more suitable for large defects and multi-fenestrated lesions (20).

Valve function preservation is the primary concern in pmVSD closure. At 1 year, no patient developed new-onset or worsening AR or TR. This favorable result likely reflects both the material properties of the bioabsorbable occluder and individualized device selection. The PDO framework has a lower elastic modulus than nitinol, theoretically exerting gentler mechanical pressure on adjacent leaflets. Furthermore, the availability of four waist-height configurations allows case-by-case optimization.

In case 1, initial use of a type II-12 occluder (5 mm waist) was associated with increased TR, presumably due to impingement on the tricuspid chordae by the longer waist. Switching to a type I-12 occluder (2.8 mm waist) restored TR to baseline. Thus, for defects close to the tricuspid septal leaflet, a shorter waist is recommended.

In case 4 (subaortic rim <3 mm), trial of a type II-10 occluder caused mild AR, likely due to left-disc compression on the right coronary cusp. Downsizing to a type II-9 occluder, combined with fine-tuning of the release axis under TEE guidance, resolved the AR. The type II (5 mm waist) was retained rather than switching to type I, based on our experience that a slightly longer waist provides additional clearance between the occluder's upper edge and the aortic leaflet, reducing the risk of leaflet entrapment. Huang et al. (21) also reported successful outcomes with bioabsorbable occluders in patients with subaortic rim <3 mm, suggesting that the device's flexibility may permit safe use in anatomies traditionally considered high-risk. Preclinical studies have demonstrated the biocompatibility of the material: Li et al. (7) showed in a canine VSD model that endothelium covered most PDO fibers by 6 months, with local inflammation confined to the occluder surface and no involvement of adjacent leaflets. Real-time TEE was invaluable in monitoring disc–valve interactions and guiding immediate adjustments (20, 22).

Complete atrioventricular block is another major complication that must be considered. Reported rates of cAVB after pmVSD closure range from 0.7% to 3.7% (4, 23, 24). In the RCT by Wang et al. (10), the bioabsorbable occluder group had a lower incidence of persistent conduction block at 24 months compared with the metal occluder group (0% vs. 11.1%, *P* = 0.036). Song et al. (11) and Huang et al. (21) reported no cAVB in their bioabsorbable occluder series. In our cohort, no patient developed cAVB, and only one had transient IRBBB that normalized within 3 months. The lower elastic modulus of the bioabsorbable material may reduce chronic mechanical compression on the conduction system (17), and gradual degradation may further mitigate the risk of local inflammation and scar formation—a trend supported by the observation that arrhythmia frequency decreased with occluder degradation in a canine model (7). However, these findings require confirmation in larger cohorts with longer follow-up.

Serial echocardiography showed progressive reduction in occluder disc area, with left-disc degradation faster than right-disc degradation, consistent with prior reports (10, 11). This difference may be explained by the higher flow velocity and pressure in the LV. Preclinical studies have shown that bioabsorbable occluders become covered by endothelial cells early after implantation, followed by gradual replacement by native fibrous tissue (7). Cong et al. (12) confirmed near-complete degradation by 2–3 years without defect recanalization. In our series, no new residual shunt appeared during follow-up, suggesting that native tissue formed an effective barrier as the occluder degraded. Complete absorption obviates the long-term risks of chronic inflammation and tissue erosion associated with permanent metal implants (25).

### 4.1 Limitations

This study has several limitations. First, the small sample size limits generalizability and statistical inference. Second, the follow-up duration (1 year) is insufficient to capture the full degradation process or late complications. Although previous studies with longer follow-up observed no recanalization after degradation, the long-term tissue repair after complete degradation requires further follow-up. Third, the lack of a control group (metal occluder or surgery) prevents direct comparison. Fourth, selection bias is possible, as only anatomically favorable cases were included. Larger, multi-center prospective studies are needed to confirm these preliminary findings.

## 5 Conclusions

TEE-guided transthoracic closure of moderate-to-large pmVSD in children using a bioabsorbable occluder appears feasible, with favorable 1-year outcomes in valvular and conduction function preservation and occluder degradation. Individualized device selection and the inherent properties of the bioabsorbable material may contribute to the favorable outcomes, although larger studies are needed to confirm long-term efficacy.

## Data Availability

The raw data supporting the conclusions of this article will be made available by the authors, without undue reservation.
